# Balancing act: Orthodontic strategies in severe periodontitis – updating clinical practice with evidence-based insights

**DOI:** 10.1097/MD.0000000000048288

**Published:** 2026-04-03

**Authors:** Gurpreet Kaur, Kulbir Kaur, Chandanpreet Kaur, Jessica Arora, Vishakha Grover, Priyanka Saluja, Suheel Manzoor Baba, Shafait Ullah Khateeb, Suraj Arora, Waled Abdulmalek Alanesi, Asmaa Ejaz Khan

**Affiliations:** a Dr. Harvansh Singh Judge Institute of Dental Sciences and Hospital, Panjab University, Chandigarh, India; b Morgan Hill Dentistry & Implants, Morgan Hill, CA; c Mike Petryk School of Dentistry, University of Alberta, Edmonton, Alberta, Canada; d Department of Restorative Dental Sciences, College of Dentistry, King Khalid University, Abha, Saudi Arabia; e Department of Operative Dentistry, Faculty of Dentistry, University of Science and Technology, Sana’a, Yemen; f Private Dental Practitioner, Dundee, UK.

**Keywords:** clinical practice, evidence based, orthodontics, severe periodontitis

## Abstract

Periodontitis is a widespread disease affecting oral structures and requiring a multidisciplinary treatment approach once it reaches its advanced form. This paper aims to provide an evidence-based update on the management of orthodontic treatment in patients with aggressive periodontics, emphasizing the need for careful assessment, timely intervention, and ongoing collaboration between orthodontists and periodontists. This article was conducted as a narrative review aimed at providing a clinically oriented synthesis of current evidence regarding orthodontic treatment in patients with stage IV periodontitis. A targeted literature search with predefined relevant key terms was performed using electronic databases, including PubMed/MEDLINE, Scopus, and Google Scholar. The search focused on English-language publications over the last 15 years. The second is a review of evidence-based studies in the combined fields of orthodontics and periodontics, with a focus on orthodontic treatment possibilities, limitations, and risks inherent in patients with periodontal disorders, particularly active periodontal disease. The evidence presented highlights that while orthodontic treatment can be beneficial for patients with severe periodontitis, it must be preceded and accompanied by thorough periodontal therapy to mitigate potential risks such as increased plaque accumulation. Overall, while challenges exist in treating these complex cases, advancements in both periodontal and orthodontic techniques offer promising avenues for improving patient outcomes.

## 1. Introduction

Periodontitis is a chronic inflammatory disease affecting tissues supporting teeth.^[1]^ Stage IV periodontitis, as defined by the 2018 European Federation of Periodontology Classification, represents the most advanced form of the disease and is characterized by severe clinical attachment loss (CAL), alveolar bone loss, and deep periodontal pockets.^[2]^ The progressive periodontal breakdown often leads to pathologic tooth migration (PTM), which in turn causes the posterior teeth to drift medially, the posterior bite to collapse, and the incisors to proclinate, rotate, and over-erupt.^[3]^ This negatively affects aesthetics, function, quality of life, and psychological well-being, frequently motivating patients to seek orthodontic care.^[4,5]^

“In order to maintain good periodontal health, it is important to have properly aligned teeth that are not occluded by traumatic means. And orthodontic forces can only be applied effectively when teeth have strong periodontal support.” This underscores the importance of orthodontic harmony for periodontal health and the need for periodontal health to achieve desired orthodontic tooth movement.^[6]^

Contemporary evidence suggests that orthodontic intervention, when carefully timed and biologically controlled, may contribute to improved occlusal stability and facilitate long-term periodontal maintenance.

This review aims to provide an evidence-based update on orthodontic management in patients with severe (stage IV) periodontitis, focusing on clinical indications, timing, biomechanics, retention, and long-term maintenance.

### 1.1. Literature search strategy

This article was conducted as a narrative review aimed at providing a clinically oriented synthesis of current evidence regarding orthodontic treatment in patients with stage IV periodontitis. A targeted literature search was performed using electronic databases, including PubMed/MEDLINE, Scopus, and Google Scholar.

The search focused on English-language publications from approximately 2010 to 2024 and included terms such as “Stage IV periodontitis,” “severe periodontitis,” “orthodontic treatment,” “pathologic tooth migration,” “periodontally compromised patients,” and “orthodontic-periodontal interdisciplinary treatment.” Reference lists of relevant articles were also screened to identify additional pertinent studies.

Priority was given to systematic reviews, clinical trials, consensus statements, and well-documented case reports with direct relevance to clinical decision-making. Given the narrative nature of this review, no formal study selection criteria or quantitative synthesis were applied. Instead, the available evidence was critically interpreted to highlight clinical indications, limitations, timing considerations, and long-term management principles for orthodontic treatment in patients with advanced periodontal disease.

## 2. Need for orthodontic therapy and treatment considerations in periodontally compromised patients

The association between malocclusion and periodontitis remains complex and multifactorial. Periodontitis is classified by stage and grade, reflecting disease severity and rate of progression, with severity influenced by CAL, probing depth, bone loss, tooth loss, and secondary occlusal trauma.

Secondary malocclusions are highly prevalent in advanced periodontitis. More than half of patients with stage III disease and over 90% with stage IV disease exhibit malocclusions, commonly including spacing, crowding, incisor protrusion, extrusion, deep bite, and crossbites.^[7,8]^ Consequently, a substantial proportion of patients with severe periodontitis meet objective criteria for orthodontic treatment, primarily due to pathological migration, occlusal dysfunction, and traumatic occlusal forces.^[5,9]^

In the absence of appropriate intervention, PTM and associated traumatic occlusion can exacerbate periodontal breakdown, increasing tooth mobility, extrusion, and attachment loss. Experimental and clinical studies have linked occlusal trauma to increased alveolar bone loss and periodontal ligament inflammation.^[4]^ Orthodontic intervention in these patients is therefore not merely elective but often essential to restore occlusal stability and facilitate long-term periodontal health as part of a multidisciplinary treatment approach.^[10,11]^

Simultaneously, the timing of orthodontic treatment is equally critical and must follow adequate control of periodontal inflammation. According to the European Federation of Periodontology guidelines, orthodontic therapy (OT) should only commence once active disease is stabilized. Certain goals must be achieved before starting OT, which include no sites with a probing pocket depth (PPD) of 5 mm accompanied by bleeding on probing, and no sites with a PPD of 6 mm or more.^[2]^ These outcomes are reached through 3 distinct phases, with the final phase implemented if the desired results were not achieved in the earlier 2 phases.^[9,12,13]^ Periodontal stabilization typically involves oral hygiene reinforcement, professional supra- and subgingival debridement, and, when indicated, adjunctive antimicrobial therapy or surgical intervention. Once the goals of active periodontal therapy are met, orthodontic treatment may be initiated approximately 3 to 6 months after nonsurgical therapy or 9 to 12 months following regenerative procedures, provided periodontal stability and patient compliance are maintained.^[9]^

Biomechanical planning in periodontally compromised patients requires careful consideration due to reduced alveolar bone support and apical displacement of the tooth’s center of resistance. As attachment loss increases, the tooth becomes more susceptible to uncontrolled tipping under conventional forces. Therefore, light, controlled forces are mandatory to prevent further attachment loss and unintended tooth movements such as extrusion.^[14]^ Tooth movements commonly required include intrusion, retraction, and correction of spacing or flaring associated with PTM.

In cases of significant bone loss, temporary anchorage devices may be advantageous to minimize unwanted reciprocal forces and preserve posterior tooth support. While fixed appliances often provide superior force control compared with removable systems, particularly for complex movements such as intrusion, appliance selection should be individualized based on periodontal status and oral hygiene capability.^[15]^ A systematic review and meta-analysis by Oikonomou and colleagues^[16]^ indicated that in the short term, patients treated with clear aligners without additional attachments demonstrated improved oral health outcomes compared with those treated with fixed appliances. However, these findings pertain to simple malocclusions and short observation periods.^[2]^

Successful outcomes in these patients depend on close collaboration between the orthodontist and periodontist, meticulous biomechanical control, and strict supportive periodontal care (SPC) throughout treatment. When appropriately planned and executed, OT is safe in patients with stabilized periodontitis, provided optimal plaque control is maintained during therapy. Notably, Kloukos et al concluded that the long-term prognosis of pathologically migrated teeth does not seem to be endangered by their orthodontic realignment, reinforcing the therapeutic value of orthodontics in restoring function and aesthetics in this patient population.^[17]^

## 3. Indications and contraindications for orthodontic treatment in patients with stage IV periodontitis

### 3.1. Indications

#### 3.1.1. After periodontal stabilization with ongoing maintenance

Orthodontic treatment is indicated only after periodontal inflammation has been controlled and the patient is enrolled in a structured SPC program; stage IV-focused guidance stresses case selection, staged care, and close perio-ortho coordination before any tooth movement.^[2,9,18–20]^

#### 3.1.2. Feasible initiation after regenerative surgery (timing evidence)

In stage IV cohorts treated regeneratively, starting orthodontic movement as early as 4 weeks post-surgery achieved periodontal outcomes comparable with 6-month initiation, provided strict SPC and hygiene were maintained.^[21]^

#### 3.1.3. Long-term stability under SPC

Retrospective stage IV data (5–12 years follow-up) show stable probing depths and radiographic bone levels when orthodontics is delivered after active periodontal therapy and maintained with individualized SPC.^[2,20]^

#### 3.1.4. Stage-IV-adapted mechanics and appliances

Because of the severely reduced periodontium, indications presume the use of modified biomechanics (light, well-controlled forces; tailored anchorage; careful appliance selection) within an interdisciplinary plan.^[2,9]^

## 4. Contraindications

### 4.1. Active periodontal disease or unstable inflammatory control

Orthodontic treatment should be deferred when periodontal inflammation is not yet controlled; stage IV guidance lists periodontal stabilization as a precondition for orthodontic movement.^[2,9,18,19]^

### 4.2. Poor adherence to SPC

Where SPC compliance is unreliable, the risk of disease progression and treatment failure increases; lack of maintenance is a practical contraindication in stage IV.^[2,9]^

### 4.3. Inability to commit to long-term retention and maintenance

Stage IV reviews emphasize lifelong retention and SPC to preserve outcomes; inability to sustain these is a relative contraindication to initiating orthodontic correction.^[2,19]^

### 4.4. High-risk systemic or local conditions as defined in stage IV trials

Stage IV randomized controlled trial protocols exclude patients with uncontrolled systemic conditions, smoking > 5 cigarettes/d, and furcation involvement of target teeth; by extension, these profiles warrant deferral or risk mitigation prior to orthodontics.^[21]^

## 5. Insights into contemporary evidence regarding orthodontic treatment in patients with periodontitis

In a recent study involving 121 patients with stage III–IV periodontitis, examining the prevalence of PTM and the need for OT identified prevalence in 74.4% and 60.3% of maxillary and mandibular teeth, respectively, with the reported need for OT in 66.1% of the cohort.^[22]^ Orthodontic treatment is often associated with mild changes in periodontal indices, such as elevation in the clinical indices of bleeding and plaque, deepening of gingival pockets, alteration in oral and subgingival microbiota, and bone loss. These changes are transient in nature as long as the prerequisites of adequate dental hygiene and inflammation control are met.^[7,23]^

Experimental and clinical evidence suggest that orthodontic treatment can be safely performed in periodontally compromised patients when periodontal inflammation is effectively controlled. Early animal studies demonstrated that complex orthodontic tooth movements, such as intrusion, can be carried out without compromising periodontal tissue integrity, even in areas with reduced periodontal support, in the absence of inflammation.^[2,13]^

These observations are further supported by evidence synthesized in a recently published systematic review by Papageorgiou and Antonoglou in 2024, including 40 studies and a total of 1608 patients with stage IV periodontitis. It established significant differences in the periodontal parameters between combined orthodontic-periodontal therapy and periodontal treatment alone, where the former revealed greater CAL gains (−0.55 mm to −0.14 mm), greater PPD reduction (−1.07 mm to −0.27 mm), greater radiographic bone loss improvement (−0.11 mm to −0.01 mm), fewer cases with III° tooth mobility (0.08–0.52), and less treatment failure (0.05–0.42).^[9,22]^

Additional support for this treatment strategy is derived from multiple case reports involving patients with periodontitis presenting with deep infrabony defects.^[6,7,12]^ In most cases, treatment protocols followed a structured, sequential approach. Initial therapy typically consisted of oral hygiene reinforcement and nonsurgical periodontal therapy, followed by reevaluation and surgical interventions such as periodontal flap surgery, if periodontal end goal was not achieved. The timing of regenerative procedures, including guided tissue regeneration and enamel matrix derivatives, varied depending on the clinical presentation.

In patients with predominantly horizontal bone defects, orthodontic treatment was often initiated prior to regenerative procedures,^[3,12,24]^ as orthodontic tooth movement facilitated the conversion of horizontal defects into narrow vertical defects, which can be easily treated with regenerative procedures, whereas the flow of treatment reversed (OT following regenerative procedure) in the presence of deep bony defects with <25% remaining bone, allowing newly formed periodontal tissues to serve as a biologic framework for subsequent tooth movement and defect stabilization.^[6]^

While appropriate treatment sequencing is essential, in patients with stage IV periodontitis undergoing orthodontic treatment, adherence to a suitable SPC regimen plays a critical role in enhancing the success of therapy. SPC recommendations vary widely when treating these patients, ranging from monthly to every 3 to 6 months.^[12,25,26]^ Studies have demonstrated that orthodontic treatment conducted under strict monthly biofilm control protocols can result in periodontal indices comparable with those observed in periodontally healthy individuals. These findings may be extended to adult patients with stage IV periodontitis. Although no universal consensus exists regarding the optimal frequency of professional oral hygiene measures, regular periodontal maintenance guided by individual risk factors, including history of smoking and diabetes, and dental risk factors, such as multiple restorations and oral hygiene maintenance routine, is strongly recommended.^[9,18,24,27]^

The growing demand for orthodontic treatment among adult patients further underscores the importance of a comprehensive periodontal evaluation prior to treatment initiation, as tooth movement in the presence of periodontitis can cause significant periodontal tissue damage. In case periodontal disease is overlooked and diagnosed later during the orthodontic treatment, the treatment should be suspended until adequate periodontal health is established by periodontal treatment.^[18]^

This clinical scenario can be better illustrated using a case report involving a 32-year-old Japanese patient who was referred for periodontal evaluation after signs of acute periodontitis were detected in the maxillary and mandibular molars during ongoing orthodontic treatment. Subsequent examination led to a diagnosis of localized severe periodontitis (stage III, grade B). OT was temporarily discontinued, followed by periodontal treatment following the given treatment plan in sequential order: scaling and root planing (SRP), full-mouth disinfection within 24 hours of SRP and adjunctive systemic antibiotic therapy for 7 days to minimize SRP-associated bacteremia, and reinforcement of oral hygiene measures. After restoration to optimum periodontal health, orthodontic treatment was resumed after 3 months, accompanied by supportive periodontal therapy and no signs of recurrence of periodontal disease were seen.^[8]^

Collectively, contemporary evidence supports the carefully controlled integration of OT into the management of advanced periodontitis. When inflammation is eliminated, risk factors are addressed, and SPC is rigorously maintained, orthodontic treatment can contribute not only to functional and aesthetic rehabilitation but also to improved periodontal stability.

## 6. Recommendation for the orthodontic management of periodontal disease patients

The purpose of this paper is to compile various published treatment plans with evidence of effects of combined orthodontic and periodontal treatment for cases with severe periodontitis. The recommended conduct of various periodontal evaluations before, during, and after orthodontic treatment would help catch periodontal destruction at its early stage and provide adequate treatment according to needs. Furthermore, this protocol recommends several courses of therapy for various periodontal diseases throughout the orthodontic treatment.^[8]^

Given that these individuals have less periodontal support, it is critical that the orthodontic appliances being utilized are as hygienic as feasible and do not interfere with good oral hygiene practices.^[4,26]^ In addition, orthodontic treatment should be completed as quickly as feasible to reduce treatment-related side effects, including root resorption and demineralization, and to prevent the patient from being exposed to extended periods of increased microbial burden. Furthermore, it is critical to understand that several systemic conditions and drugs taken by adults with stage IV periodontitis may alter the dynamics of tooth movement. As a result, careful patient monitoring is required in order to give tailored care.^[9,14]^

When should orthodontic treatment of periodontal patients begin?^[9]^

Once periodontal inflammation has subsided:

- Step 1 of periodontal therapy – patient education and oral hygiene instructions.
- Step 2 – supragingival, subgingival calculus removal.
- Periodontal treatment response evaluation.
- Step 3 – initiated for debridement of deep pockets, furcation, and infrabony defects regeneration.

The goal of periodontal therapy is to achieve the following endpoints:

- No pockets >5 mm with bleeding on probing.
- No pockets >6 mm.

Once endpoints are achieved, patients are placed in SPC.

Orthodontic treatment begins once periodontal tissues have adequately healed:

- Three to 6 months following either surgical or nonsurgical periodontal therapy.
- Nine to 12 months following regenerative surgery treatments.

The impacts of OT on the health of periodontal tissues are the following:

- Rise in the bleeding/plaque indices in patients.
- Gingival pocket deepening.
- Slight bone loss.
- Alterations in the oral and subgingival microbiota, either in terms of quantity or quality.

Periodontal side effects associated with OT are minimal if proper oral hygiene and inflammation management are maintained. Although it is believed that removable orthodontic equipment reduces plaque buildup, most orthodontic appliances can support periodontal health when used in conjunction with appropriate oral hygiene practices.^[8,9,12]^

## 7. Retention, relapse, and long-term maintenance

Retention in stage IV periodontitis patients requires a careful balance between mechanical stability and periodontal health. Fixed retainers are often indicated in cases with extensive bone loss, where they serve not only to maintain alignment but also act as periodontal splints. Flexible lingual retainers are widely used because they permit physiological tooth movement and do not depend on patient compliance, although they carry a higher risk of plaque accumulation and require meticulous hygiene.^[2,3,9]^ Removable retainers, by contrast, facilitate hygiene and allow easier monitoring of periodontal status, but their effectiveness depends heavily on patient compliance.^[13]^ In many cases, dual retention combining a fixed retainer with a vacuum-formed removable appliance worn at night offers added protection against relapse.^[9]^ Ultimately, clinical decision-making should be individualized, taking into account patient preference, oral hygiene capacity, and the initial treatment plan (see Table 1).

**Table 1:** Comparison of retainer types in stage IV periodontitis patients.

Retainer type	Advantages	Limitations	Key references
Fixed (flexible lingual)	Superior stability; acts as periodontal splint; no compliance needed	Higher plaque accumulation risk; requires meticulous hygiene	Garbo et al,^2^ Papageorgiou et al,^9^ Ramachandra et al^3^
Removable (vacuum-formed)	Easier hygiene; allows monitoring	Compliance-dependent; less effective alone	Levin et al^[13]^
Dual retention (fixed + removable)	Combines stability with hygiene monitoring; nighttime wear reduces relapse	Requires patient cooperation; more complex	Papageorgiou et al^[9]^

Retainer choice should be individualized based on patient preference, oral hygiene, and periodontal condition.

Relapse remains a significant concern in patients with reduced periodontal support, as alveolar bone loss compromises anchorage and increases the likelihood of occlusal changes.^[9]^ Extended retention periods are often necessary, particularly following procedures such as de-rotation or space closure, which carry a higher relapse risk.^[28]^ Supportive periodontal therapy plays a critical role in minimizing relapse. A systematic review by Erbe et al confirmed that regular supportive care prevents the negative impacts of orthodontic treatment on an already impaired periodontium.^[4,5]^ Importantly, comparative evidence highlights the added value of orthodontic intervention in stage IV periodontitis patients. Zasčiurinskienė et al^[26]^ reported that relapse rates were significantly higher in patients treated with periodontal therapy alone (≈33% after 2 years), whereas those who underwent combined periodontal and OT experienced substantially lower relapse rates (≈15%). This finding underscores that orthodontic treatment, when integrated with supportive care, enhances long-term stability compared with periodontal therapy alone (see Table 2).

**Table 2:** Relapse rates with and without orthodontic therapy in stage IV periodontitis patients.

Treatment approach	Reported relapse rate	Reference
Periodontal therapy alone	≈33% after 2 yr	Zasčiurinskienė et al^[26]^
Combined perio-ortho therapy	≈15% after 2 yr	Zasčiurinskienė et al^[26]^

Comparative evidence highlights the added value of orthodontic intervention in reducing relapse rates when combined with supportive care.

Long-term success depends on integrating orthodontic retention with SPC. Maintenance visits every 3 to 4 months are recommended for professional cleaning and monitoring of attachment levels.^[13,17]^ Classic evidence from Ericsson et al^[29]^ demonstrated that orthodontic forces applied to a plaque-free, but periodontally impaired periodontium did not cause infrabony defects, whereas the presence of plaque led to attachment loss, underscoring the importance of hygiene. Patient education and adherence to retainer use remain vital,^[30]^ and collaboration between orthodontists and periodontists ensures that retention strategies adapt to evolving periodontal conditions.^[31]^

True stability in periodontally compromised patients is not solely mechanical but biological. Case reports have shown that interdisciplinary management can preserve teeth with severe mobility when retention is combined with periodontal therapy.^[12,32]^ Long-term follow-up studies confirm that stability is achievable, but only with continuous supportive care and flexible retention protocols.^[10,23]^ As Fleming and Andrews^[33]^ emphasize, retention in periodontitis patients must be adaptive, integrating biological monitoring with mechanical retention to ensure sustained outcomes.

## 8. Limitations and future directions

There are a number of restrictions on this narrative review. The inclusion of studies with different designs, classification terminology, follow-up durations, and treatment procedures reduces generalizability, and the lack of a systematic screening protocol creates selection bias. There is a lack of long-term prospective data on orthodontic outcomes in patients with stage IV periodontitis, and much of the material is from before the 2017 periodontal categorization, making direct comparison a challenge. Standardized outcome indicators, such as levels of radiographic bone, levels of clinical attachment, and patient-reported outcomes, should be prioritized in future studies. Periodontal stability and tooth survival after therapy can only be determined with long-term follow-up investigations. Digital planning tools, artificial intelligence, and cone beam computed tomography imaging have all made great strides recently, which bodes well for better risk categorization. To best serve this complicated group of patients, it will be essential to develop interdisciplinary recommendations based on consensus and investigate how systemic comorbidities affect orthodontic tooth mobility.

## 9. Conclusion

Orthodontic treatment in patients with stage IV periodontitis is feasible and can improve functional stability, aesthetics, and long-term periodontal health when delivered within a carefully coordinated interdisciplinary framework. Successful outcomes depend on prior periodontal stabilization, biologically controlled light forces, individualized biomechanics, and strict SPC throughout treatment and retention. Current evidence suggests that combined orthodontic-periodontal therapy offers greater clinical stability and reduced relapse than periodontal therapy alone, although outcomes remain highly patient-dependent and require lifelong maintenance. Overall, contemporary advances support the cautious but effective integration of orthodontics into the rehabilitation of patients with advanced periodontal disease.

## Author contributions

**Conceptualization:** Gurpreet Kaur.

**Data curation:** Gurpreet Kaur, Priyanka Saluja.

**Formal analysis:** Kulbir Kaur, Suheel Manzoor Baba.

**Investigation:** Kulbir Kaur, Shafait Ullah Khateeb.

**Methodology:** Chandanpreet Kaur.

**Project administration:** Chandanpreet Kaur.

**Resources:** Jessica Arora, Asmaa Ejaz Khan.

**Software:** Jessica Arora, Asmaa Ejaz Khan.

**Supervision:** Vishakha Grover.

**Validation:** Vishakha Grover.

**Visualization:** Suheel Manzoor Baba, Shafait Ullah Khateeb.

**Funding acquisition:** Suraj Arora.

**Writing – original draft:** Priyanka Saluja.

**Writing – review & editing:** Waled Abdulmalek Alanesi, Asmaa Ejaz Khan.

## References

[1] Tonetti MS Greenwell H Kornman KS. Staging and grading of periodontitis: framework and proposal of a new classification and case definition. J Clin Periodontol. 2018;45(Suppl 20):S149–61. 29926495 10.1111/jcpe.12945

[2] Garbo D Baima G Mariani GM Romano F Aimetti M. Orthodontic treatment in stage IV periodontitis patients: timing, management and long-term prognosis. Semin Orthod. 2024;30:113–22.

[3] Ramachandra C Shetty PC Rege S Shah C. Ortho-perio integrated approach in periodontally compromised patients. J Indian Soc Periodontol. 2011;15:414–7. 22368371 10.4103/0972-124X.92583 PMC3283944

[4] Erbe C Heger S Kasaj A Berres M Wehrbein H. Orthodontic treatment in periodontally compromised patients: a systematic review. Clin Oral Investig. 2023;27:79–89. 10.1007/s00784-022-04822-1 PMC9877066 36502508

[5] Zasciurinskiene E Lindsten R Slotte C Bjerklin K. Orthodontic treatment in periodontitis-susceptible subjects: a systematic literature review. Clin Exp Dent Res. 2016;2:162–73. 29744163 10.1002/cre2.28 PMC5839229

[6] Jiang K Jiang LS Li HX Lei L. Periodontal-orthodontic interdisciplinary management of a “periodontally hopeless” maxillary central incisor with severe mobility: a case report and review of literature. World J Clin Cases. 2022;10:4550–62. 35663057 10.12998/wjcc.v10.i14.4550 PMC9125257

[7] Deepa D Mehta D Puri VK Shetty S. Combined periodontic-orthodontic-endodontic interdisciplinary approach in the treatment of periodontally compromised tooth. J Indian Soc Periodontol. 2010;14:139–43. 21691554 10.4103/0972-124X.70837 PMC3110470

[8] Morikawa S Watanabe K Otsuka R Asoda S Nakagawa T. Periodontal therapy for localized severe periodontitis in a patient receiving fixed orthodontic treatment: a case report. J Med Case Rep. 2023;17:19. 36658639 10.1186/s13256-023-03751-1 PMC9854180

[9] Papageorgiou SN Antonoglou GN Eliades T Martin C Sanz M. Orthodontic treatment of patients with severe (stage IV) periodontitis. Semin Orthod. 2024;30:123–34.

[10] Boyer S Fontanel F Danan M Olivier M Bouter D Brion M. Severe periodontitis and orthodontics: evaluation of long-term results. Int Orthod. 2011;9:259–73. 21855438 10.1016/j.ortho.2011.06.004

[11] Carvalho CV Saraiva L Bauer FPF. Orthodontic treatment in patients with aggressive periodontitis. Am J Orthod Dentofacial Orthop. 2018;153:550–7. 29602347 10.1016/j.ajodo.2017.08.018

[12] Ishihara Y Tomikawa K Deguchi T. Interdisciplinary orthodontic treatment for a patient with generalized aggressive periodontitis: assessment of IgG antibodies to identify type of periodontitis and correct timing of treatment. Am J Orthod Dentofacial Orthop. 2015;147:766–80. 26038081 10.1016/j.ajodo.2014.09.022

[13] Levin L Einy S Zigdon H Aizenbud D Machtei EE. Guidelines for periodontal care and follow-up during orthodontic treatment in adolescents and young adults. J Appl Oral Sci. 2012;20:399–403. 23032199 10.1590/S1678-77572012000400002 PMC3881826

[14] Gyawali R Bhattarai B. Orthodontic management in aggressive periodontitis. Int Sch Res Notices. 2017;2017:8098154. 28299350 10.1155/2017/8098154 PMC5337368

[15] Han L. Advances in the study of the relationship between orthodontics and periodontitis. Trans Mater Biotechnol Life Sci. 2024;2:54–61.

[16] Oikonomou E Foros P Tagkli A Rahiotis C Eliades T Koletsi D. Impact of aligners and fixed appliances on oral health during orthodontic treatment: a systematic review and meta-analysis. Oral Health Prev Dent. 2021;19:659–72. 34874143 10.3290/j.ohpd.b2403661 PMC11641221

[17] Kloukos D Roccuzzo A Stähli A Sculean A Katsaros C Salvi GE. Effect of combined periodontal and orthodontic treatment of tilted molars and of teeth with intra-bony and furcation defects in stage IV periodontitis patients: a systematic review. J Clin Periodontol. 2022;49:121–48. 34761413 10.1111/jcpe.13509

[18] Chackartchi T Polak D Stabholz A Chaushu S. Orthodontic treatment in periodontitis patients [published online ahead of print June 26, 2025]. Periodontol 2000. doi: 10.1111/prd.12634. 10.1111/prd.12634 40569018

[19] Longlax MC Huertas ZM Monroy GJ Guerrero MG. Orthodontic and periodontal treatment in generalized stage IV periodontitis: a case report. Rev Estomatol. 2025;33.

[20] Garbo D Aimetti M Bongiovanni L. Periodontal and orthodontic synergy in the management of stage IV periodontitis: challenges, indications and limits. Life (Basel). 2022;12:2131. 36556496 10.3390/life12122131 PMC9782082

[21] Jepsen K Tietmann C Kutschera E. The effect of timing of orthodontic therapy on the outcomes of regenerative periodontal surgery in patients with stage IV periodontitis: a multicenter randomized trial. J Clin Periodontol. 2021;48:1282–92. 34312872 10.1111/jcpe.13528

[22] Zhong W Zhou C Yin Y. Expert consensus on orthodontic treatment of patients with periodontal disease. Int J Oral Sci. 2025;17:27. 40175337 10.1038/s41368-025-00356-w PMC11965299

[23] Tietmann C Jepsen S Kauer R Jepsen K. Clinical effectiveness of regenerative periodontal surgery and orthodontic tooth movement with clear aligners in stage IV periodontitis: a case series. Quintessence Int. 2024;55:348–57. 38619257 10.3290/j.qi.b5213521

[24] Panwar M Jayan B Arora V Singh S. Orthodontic management of dentition in patients with periodontally compromised dentition. J Indian Soc Periodontol. 2014;18:200–4. 24872629 10.4103/0972-124X.131325 PMC4033887

[25] Khatri A Khatri M Bansal M Batra P Aziz SB. Periodontal and microbiological evaluation in cleft lip/palate patients undergoing orthodontic treatment: a cross-sectional study. J Periodontol. 2025;96:44–54. 38924066 10.1002/JPER.24-0085

[26] Zasčiurinskienė E Bulotaitė S Bjerklin K Lodienė G Šidlauskas A Zaborskis A. Knowledge, attitudes, and interest in orthodontic treatment: a cross-sectional study in adults with stage III-IV periodontitis and secondary malocclusions. BMC Oral Health. 2023;23:853. 37951899 10.1186/s12903-023-03605-8 PMC10640755

[27] Lim BH. Orthodontic treatment in a patient with severe periodontitis. Int Dent J. 2024;74:S341.

[28] Vinod K Reddy YG Reddy VP Nandan H Sharma M. Orthodontic–periodontics interdisciplinary approach. J Indian Soc Periodontol. 2012;16:11–5. 22628956 10.4103/0972-124X.94597 PMC3357017

[29] Ericsson I Tehlander B Lindhe J Okamoto H. The effect of orthodontic tilting movements on the periodontal tissues of infected and non‑infected dentitions in dogs. J Clin Periodontol. 1977;4:278–93. 271655 10.1111/j.1600-051x.1977.tb01900.x

[30] Gehlot M Sharma R Tewari S Kumar D Gupta A. Effect of orthodontic treatment on periodontal health of periodontally compromised patients. Angle Orthod. 2022;92:324–32. 34882193 10.2319/022521-156.1 PMC9020398

[31] Chaushu S. Exploring the symbiotic relationship between orthodontics and periodontics. Semin Orthod. 2024;30:77–9.

[32] Carnio J Casarin M Carnio JK Pirih FQ. A successful periodontal-orthodontic treatment of an advanced case of dental trauma in a 9-year-old patient: a case report with 16-year follow-up [published online ahead of print February 28, 2026]. Clin Adv Periodontics. doi: 10.1002/cap.70047. 10.1002/cap.70047 PMC13603041 41761936

[33] Fleming PS Andrews J. Periodontitis: orthodontic implications and management. Br Dent J. 2024;237:334–40. 39271869 10.1038/s41415-024-7789-6 PMC11399084

